# Attraction of Female *Aedes aegypti* (L.) to Aphid Honeydew

**DOI:** 10.3390/insects10020043

**Published:** 2019-02-01

**Authors:** Daniel A. H. Peach, Regine Gries, Nathan Young, Robyn Lakes, Erin Galloway, Santosh Kumar Alamsetti, Elton Ko, Amy Ly, Gerhard Gries

**Affiliations:** Department of Biological Sciences, Faculty of Science, Simon Fraser University, Burnaby, BC V5A 1S6, Canada; mgries@sfu.ca (R.G.); nathan_young@sfu.ca (N.Y.); robyn_lakes@sfu.ca (R.L.); eqgallow@sfu.ca (E.G.); Santosh_kumar@sfu.ca (S.K.A.); elton_ko@sfu.ca (E.K.); lyamyl@sfu.ca (A.L.); gries@sfu.ca (G.G.)

**Keywords:** *Aedes aegypti*, *Acyrthosiphon pisum*, *Myzus persicae*, *Vicia faba*, honeydew, honeydew odorants, mosquito sugar feeding, microbe-emitted odorants, mosquito olfaction

## Abstract

Plant sugar is an essential dietary constituent for mosquitoes, and hemipteran honeydew is one of the many forms of plant sugar that is important to mosquitoes. Many insects rely on volatile honeydew semiochemicals to locate aphids or honeydew itself. Mosquitoes exploit volatile semiochemicals to locate sources of plant sugar but their attraction to honeydew has not previously been investigated. Here, we report the attraction of female yellow fever mosquitoes, *Aedes aegypti*, to honeydew odorants from the green peach aphid, *Myzus persicae*, and the pea aphid, *Acyrthosiphon pisum*, feeding on fava bean, *Vicia faba*. We used solid phase micro-extraction and gas chromatography-mass spectrometry to collect and analyze headspace odorants from the honeydew of *A. pisum* feeding on *V. faba*. An eight-component synthetic blend of these odorants and synthetic odorant blends of crude and sterile honeydew that we prepared according to literature data all attracted female *A. aegypti*. The synthetic blend containing microbial odor constituents proved more effective than the blend without these constituents. Our study provides the first evidence for anemotactic attraction of mosquitoes to honeydew and demonstrates a role for microbe-derived odorants in the attraction of mosquitoes to essential plant sugar resources.

## 1. Introduction

Honeydew is a sugar-rich liquid [1] secreted by aphids and scale insects feeding on plant sap [2]. Honeydew may be available at times or in locations when other sources of sugar, such as floral nectar, are not available or abundant. Many insects feed on honeydew, including honey bees, ants, wasps [1,2], and even blood-feeding dipterans such as deer flies [3,4], black flies [5,6], sand flies [7], and mosquitoes [8,9,10,11].

Plant sugar is an essential basic food for adult male and female mosquitoes [12]. Mosquito populations can persist only through ready access to plant sugar, even if they have ready access to blood [13]. Newly eclosed mosquitoes survive for only a few days without sugar [12], and sugar deprivation severely constrains the ability of mosquito males to inseminate females [12,13]. Plant sugar provides energy to male and female mosquitoes for mating and blood-feeding, and originates energy stores for overwintering females [12]. Most of the ingested plant sugar is stored in the crop, where it can be metabolized quickly to provide energy for flight [12,14], with the excess converted into glycogen or lipid for storage [12,14,15]. Newly-eclosed mosquito females are low in energy reserves [16] and preferentially seek sources of plant sugar rather than vertebrate blood [16,17,18,19]. Plant sugar also enhances the vectorial capacity of mosquitoes [20,21]. Mosquitoes feed on many forms of plant sugar including floral and extra-floral nectar, fruit juices, exudate from damaged plant tissue, plant sap they access with their piercing mouthparts [12], honeydew [8,9,10,11], and even ant regurgitate [22]. Most mosquitoes extensively exploit floral nectar but also use honeydew when nectar is scare, as do other insects [23]. For some mosquitoes, honeydew provides a valuable primary plant sugar source [11].

Inflorescence odorants are the most important cues that guide mosquitoes to floral nectar [12,24,25]. Numerous floral and fruit odorants have been identified and eventually may be used for monitoring or controlling mosquito populations, but no study has yet addressed whether mosquitoes are attracted to honeydew. Many insects that feed on honeydew, or that consume or parasitize the hemipteran insects that produce it, are attracted to honeydew odorants [26,27,28]. This may also apply to mosquitoes.

Aphid honeydew and floral nectar contain sugars and amino acids [1,29,30] that exogenous microbes metabolize, producing odorants in the process [31,32,33,34]. Mosquitoes respond to microbial odorants when they forage for hosts [35,36,37,38], and seek oviposition sites [39]. Microbial odorants emanating from aphid honeydew attract aphidophagous hoverfly predators [32] and have been speculated to attract mosquitoes [40].

The yellow fever mosquito, *Aedes aegypti*, is a widely distributed mosquito that can vector many arboviruses including dengue, yellow fever, chikungunya, and Zika [41,42,43,44]. In the laboratory, *Ae. aegypti* have been observed to imbibe honeydew from pea aphids, *Acyrthosiphon pisum*, and green peach aphids, *Myzus persicae,* colonizing broad beans, *Vicia faba* (DP, pers. obs.). Working with broad bean-colonizing pea and green peach aphids and *Ae. aegypti* as model organisms, we tested the hypothesis that *Ae. aegypti* females are attracted to (i) natural aphid honeydew odorants, (ii) a synthetic blend of these odorants, and (iii) the microbe-produced constituents of this blend.

## 2. Materials and Methods

### 2.1. Rearing of Experimental Mosquitoes

We reared mosquitoes in the insectary of Simon Fraser University (SFU) at temperatures of 23–26 °C, a photoperiod of 14L:10D, and a 40–60% RH. We maintained adult mosquitoes in mesh cages (30 × 30 × 46 cm high) and provisioned them *ad libitum* with a 10% sucrose solution. Once a week, DP fed female mosquitoes on his arm (SFU’s Office of Research Ethics advised ethics approval is not required), 3 days later giving them access to a water-containing 354 mL cup (Solo Cup Comp., Lake Forest, IL, USA) with a paper towel (Kruger Inc., Montreal, QC, Canada) lining its sides. We transferred strips of paper towel carrying *Ae. aegypti* eggs into a small circular glass dish (10 cm diameter × 5 cm high), filled with water and inoculated with brewer’s yeast (U.S. Biological Life Sciences, Salem, MA, USA). Upon larval hatching (2–4 days later), we transferred the larvae with the water to water-filled trays (45 × 25 × 7 cm high) and provisioned them with NutriFin Basix tropical fish food (Rolf C Hagen Inc., Montreal, QC, Canada). Daily, we transferred pupae via a 7-mL plastic pipette (VWR International, Radnor, PA, USA) to water-containing 354-mL Solo cups (Solo Cup Comp.) covered with a mesh lid. We aspirated eclosed adults into separate Solo cups, fitted with a cotton ball soaked in a 10% sucrose solution. Simon Fraser University’s Office of Research Ethics advised that ethics approval is not required for this study.

### 2.2. Rearing of Plants and Aphids

We grew fava beans from seed (Northwestern Seeds, Vernon, BC, Canada) in a greenhouse at SFU (Burnaby, BC, Canada) under a 16L:10D light regime, watering plants every other day. We kept colonies of green peach aphids and pea aphids on fava bean plants in separate bug dorms (61 × 61 × 61 cm) (BioQuip Products, Rancho Dominguez, CA, USA) under these same conditions.

### 2.3. General Design of Y-Tube Behavioural Experiments

To determine whether mosquitoes are attracted to aphid-infested or mechanically injured plants, we ran bioassays in Y-tube olfactometers (diameter: 2.5 cm; length of the main and lateral arms: 23 cm and 19 cm, respectively; angle of lateral arms: 120°) inclined at 45° [45]. We placed the treatment and the control stimulus (e.g., a plant with or without aphid infestation) in a plastic oven bag (Reckitt Benckiser Inc., Mississauga, ON, Canada) and tightly connected the bag to a randomly assigned lateral arm of the Y-tube. A carbon filter affixed to a small opening in one corner of each bag allowed us to draw purified air through the bags and the Y-tube. For each bioassay, we placed a single, 1–to 3-day-old, 24-h sugar-deprived female mosquito into a holding glass tube (diameter: 2.5 cm; length: 26 cm) with stainless steel mesh covering both openings. We bioassayed young mosquitoes (which are hardly responsive to vertebrate host cues [17,18,19]) to minimize behavioral effects of olfactory cues associated with the observer. To commence a bioassay, we then attached the holding tube to the Y-tube stem via a ground glass joint. Following a 60-s acclimation period, we removed the wire mesh and initiated airflow at a rate of 4 cm s^−1^ via a mechanical pump, thus carrying volatiles towards the mosquito that could now enter the Y-tube. For each replicate, we employed a clean Y-tube, a new female mosquito, and new test stimuli. We recorded the lateral arm of the Y-tube that a mosquito entered first, and considered all mosquitoes making no decisions within 5 min as non-responders, which we excluded from the statistical analyses.

### 2.4. Attractiveness of Aphid-Infested and Honeydew-Soiled Plants

We assigned potted bean plants with 6–10 “true” leaves to a treatment or a control group and placed them in separate plastic cages (21 × 26 × 32 cm). We released 20 green peach aphids or 20 pea aphids onto treatment plants but not control plants, allowing honeydew to accumulate on treatment plants over seven days. Over this time, colonies of green peach aphids and pea aphids grew to a mean size of 31 and 103 individuals, respectively. To account for the possibility that mechanical injury-related plant odorants in addition to honeydew odorants affect the mosquitoes’ responses, we mechanically injured each plant [46] by cutting one leaf along its long axis and then left the plant for 1 h prior to commencing a bioassay. In Y-tube olfactometers, we offered mosquitoes a choice between two mechanically injured bean plants (each inside an oven bag) that we had infested, or not (control), with either green peach aphids (Exp. 1) or pea aphids (Exp. 2) (Table 1).

### 2.5. Attractiveness of Mechanically-Injured Plants

To determine whether plant odorants derived from mechanical injury suffice to attract mosquitoes, we mechanically injured plants (see above), and in Y-tube olfactometers offered mosquitoes a choice between two non-infested bean plants (each inside an oven bag) that we had, or had not (control) mechanically injured (see above) (Table 1, Exp. 3).

### 2.6. Attractiveness of Plants in the Presence of Non-Feeding Aphids

To separate the effects of aphid feeding and aphid presence on the attraction of mosquitoes, we offered mosquitoes a choice between two intact bean plants (each inside an oven bag) that we paired with a mesh-covered Petri dish containing, or not (control), 100 non-feeding pea aphids (Table 1, Exp. 4).

### 2.7. Honeydew Collection and Odorant Analysis

We collected (commonly discoloured) droplets of honeydew from plants heavily infested with pea aphids, using a 10-µL glass capillary fitted with a rubber bulb. We collected a total of 50 µL of honeydew and expelled it into a 4-mL glass vial with a rubber septum lid. Through this lid, we inserted a carboxen-polydimethylsiloxene-coated solid-phase micro extraction (SPME) fibre (75 µm; Supelco Inc., Bellefonte, PA, USA), allowing absorption of honeydew odorants on this fibre for 24 h at room temperature. Prior to each odorant collection, we conditioned the fibre at 280 °C for 5 min in a gas chromatograph (GC) injection port. We desorbed odorants from the fibre in the hot (250 °C) injection port of the GC, and analyzed odorants by GC-mass spectrometry (MS) using a Saturn 2000 Ion Trap GC-MS fitted with a DB-5 GC-MS column (30 m × 0.25 mm i.d.; Agilent Technologies Inc., Santa Clara, CA, USA) in full-scan electron impact mode. We used a flow of helium (35 cm s^−1^) as the carrier gas with the following temperature program: 40 °C (5 min), 10 °C min^−1^ to 280 °C (held for 10 min). We identified volatiles by comparing their retention indices (RI) relative to n-alkane standards [47] and their mass spectra with those reported in the literature [48] and with those of authentic standards.

### 2.8. Preparation and Testing of Synthetic Honeydew Odorant Blends

We prepared three blends of synthetic honeydew odorants. Two blends reflected the composition of crude honeydew collected and analyzed in this study (CHD_1_), and in a previous study (CHD_2_) [32] (Table 2), and a third blend resembled the composition of sterilized honeydew (SHD), as previously reported [32] (Table 2) for anemotactic attraction of mosquitoes in paired-trap experiments. We dissolved all blends in a 1-mL mixture of pentane (50%) and ether (50%), and pipetted treatment and corresponding solvent control stimuli into separate 4-mL glass vials with a 2-mm hole in the lid. We tested the CHD_1_ at doses equivalent to 2.5 × 10^1^ µL and 2.5 × 10^0^ µL of crude honeydew (Exps. 5,6), the CHD_2_ at honeydew equivalent doses of 2.5 × 10^6^ μL, 2.5 × 10^5^ μL, 2.5 × 10^4^ μL, 2.5 × 10^3^ μL, 2.5 × 10^1^ µL, and 2.5 × 10^0^ µL (Exps. 8–15), and the SHD at honeydew equivalent doses of 2.5 × 10^6^ μL and 2.5 × 10^5^ μL (Exps. 7, 14, 15). The dose equivalents tested in our bioassays are biologically relevant, considering that 2.5 × 10^1^ µL of honeydew is approximately the amount of honeydew produced by 25 pea aphids per day [49] and that aphid infestations can reach several thousand individuals per m^2^ [50,51].

### 2.9. Captures of Mosquitoes in Traps Baited with Synthetic Honeydew Odorant Blends

In laboratory mesh-cage experiments, we tested captures of mosquitoes in traps baited with synthetic honeydew odorant blends (see below). Each cage (77 × 78 × 104 cm) was wrapped with black cloth except for the top, allowing light entry from above. We provided illumination with a shop light housing (Lithonia Lighting, Atlanta, GA, USA) fitted with two conventional 1.22-m fluorescent tubes (F32T8/T1835 Plus, Phillips, Amsterdam, The Netherlands). The cage housed two burette stands separated by 25 cm, each stand carrying a Delta trap 50 cm above the cage floor [52]. We prepared traps from white cardstock (71.28 × 55.88 cm) (Staples Inc., Farmingham, MA, USA; ACCO Brands Corp., Lake Zurich, IL, USA) that we cut to size (15 × 30 cm), coated with adhesive (The Tanglefoot Company, Marysville, OH, USA) on the inside, and then folded into a Delta-type trap (15 × 9 × 8 cm high). We randomly assigned the treatment and control stimuli (see below) to one trap in each pair. For each bioassay replicate, we released 50 1–3-day-old, 24-h sugar-deprived females from a Solo cup (see above) into a cage and recorded trap captures 24 h later. We ran experiments at 23–26 °C, 40–60% RH, and a photoperiod of 14L:10D, commencing the bioassay 4–6 h prior to onset of the scotophase.

We dissolved all synthetic honeydew blends in a 1-mL mixture of pentane (50%) and ether (50%), pipetted treatment and solvent control stimuli into separate 4-mL glass vials with a 2-mm hole in the lid, and randomly assigned the treatment and the control vials to one trap in each pair. We tested the CHD_1_ at a dose of 2.5 × 10^1^ µL honeydew equivalents (Exp. 5), and the CHD_2_ at doses of 2.5 × 10^6^ μL, 2.5 × 10^5^ μL, 2.5 × 10^4^ μL, 2.5 × 10^3^ μL, and 2.5 × 10^1^ µL honeydew equivalents (Exps. 6–10). To compare the relative attractiveness of crude and sterilized honeydew, we tested the CDV_2_ vs. the SHD at doses of 2.5 × 10^6^ μL and 2.5 × 10^5^ μL honeydew equivalents (Exps. 11, 12).

### 2.10. Statistical Analyses

We analyzed behavioral data using SAS statistical software version 9.4 (SAS Institute Inc., Cary, NC, USA), excluding experimental replicates with no mosquitoes responding. We analyzed data from Y-tube olfactometer experiments (Exps. 1–4) using a two-tailed exact-goodness-of-fit test. For cage experiments 5–15, we compared the mean proportions of responders to paired test stimuli using a binary logistic regression model and worked with back-transformed data to obtain means and confidence intervals.

## 3. Results

### 3.1. Attractiveness of Plants that Were Aphid-Infested, Mechanically Injured, or Paired with Non-Feeding Aphids

**Figure 1:**
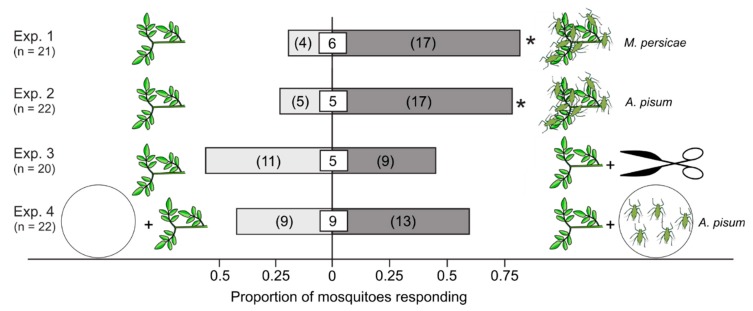
Proportion of female yellow fever mosquitoes, *Aedes aegypti*, responding in binary choice Y-tube olfactometer experiments (N = 20–22 replicates) to fava bean plants, *Vicia faba*, that were non-infested (control) or that were (*i*) infested with green peach aphids, *Myzus persicae* (Exp. 1), or pea aphids, *Acyrthosiphon pisum* (Exp. 2); (*ii*) mechanically injured (Exp. 3), or (*iii*) paired with 100 non-feeding pea aphids. Numbers in parentheses represent the number of mosquitoes selecting a test stimulus, and numbers in square boxes in bars represent the number of non-responding mosquitoes. For each experiment, an asterisk (*) indicates a significant preference for a test stimulus (*p* < 0.05; exact test of goodness-of-fit).

In Y-tube olfactometer experiments, plants infested with green peach aphids (Exp. 1) or pea aphids (Exp. 2) attracted 81% and 77.3% of responding mosquitoes, respectively, significantly more than aphid-free control plants (Exp. 1: z = −2.84, *p* = 0.007; Exp. 2: z = −2.56, *p* = 0.017; Figure 1). Intact and mechanically injured plants were equally attractive to female mosquitoes (z = 0.45, *p* = 0.82; Figure 1, Exp. 3), as were intact plants in the presence or absence of non-feeding pea aphids (z = -0.85, *p* = 0.52) (Figure 1, Exp. 4).

### 3.2. Analyses of Honeydew Headspace Odorants

**Figure 2:**
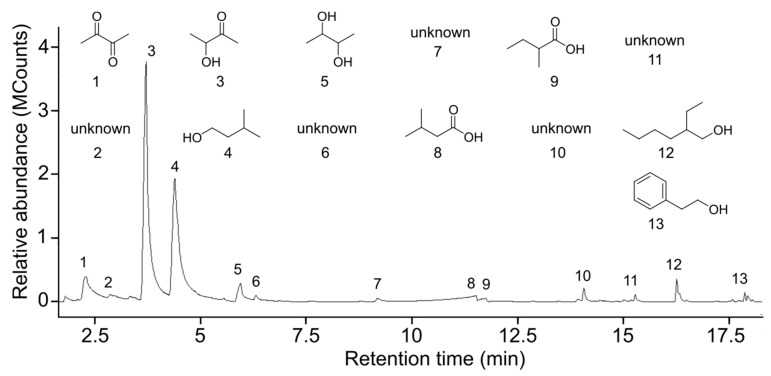
Total ion chromatogram of pea aphid honeydew odorants collected on, and thermally desorbed from, a solid-phase micro extraction (SPME) fibre. Compound identity is as follows: 1 = butanedione; 2 = unknown; 3 = 3-hydroxybutanone; 4 = 3-methylbutan-1-ol; 5 = 2,3-butanediol; 6 = unknown; 7 = unknown; 8 = 3-methylbutanoic acid; 9 = 2-methylbutanoic acid; 10 = unknown; 11 = unknown; 12 = 2-ethylhexanol; 13 = 2-phenylethanol.

Desorption and GC-MS analyses of SPME collected honeydew headspace odorants consistently revealed eight compounds (Figure 2 and Table 1), including ketones, alcohols, acids, and aldehydes. The most abundant compounds were 3-hydroxybutanone and 3-methyl-1-butanol.

### 3.3. Attractiveness of Synthetic Honeydew Odorant Blends in Y-tube Olfactometers

**Figure 3:**
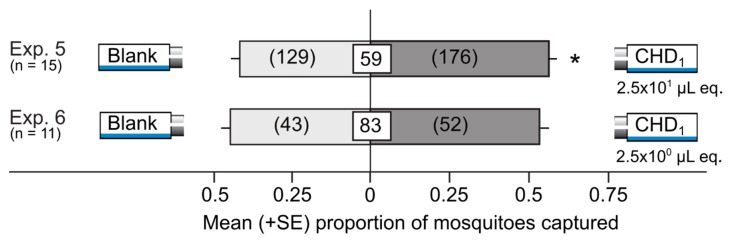
Mean proportion (+SE) of female yellow fever mosquitoes, *Aedes aegypti*, captured in Experiments 5 and 6 in paired traps that were baited with the CHD_1_ (a synthetic blend of crude pea aphid honeydew odorants prepared according to our own data; Figure 2 and Table 2) or fitted with a corresponding solvent (blank) control. Numbers in parentheses represent the total number of mosquitoes selecting a test stimulus, and numbers within white squares indicate the mean percentage of mosquitoes not captured (non-responders). An asterisk (*) indicates a significant preference for a test stimulus (*p* < 0.05; binary logistic regression). The dose of 2.5 × 10^1^ µL equivalents (eq.) of honeydew approximates the amount of honeydew produced by 25 pea aphids per day [41].

The CHD_1_ (a synthetic blend of crude honeydew odorants prepared according to our own data; Figure 2) at a dose of 2.5 × 10^1^ µL honeydew equivalents (Exp. 5), but not at a dose of 2.5 × 10^0^ µL honeydew equivalents (Exp. 6), attracted significantly more mosquitoes than the corresponding solvent control stimuli (Exp. 5: z = 2.7, *p* = 0.007; Exp. 6: z = 0.92, *p* = 0.36; Figure 3).

**Figure 4:**
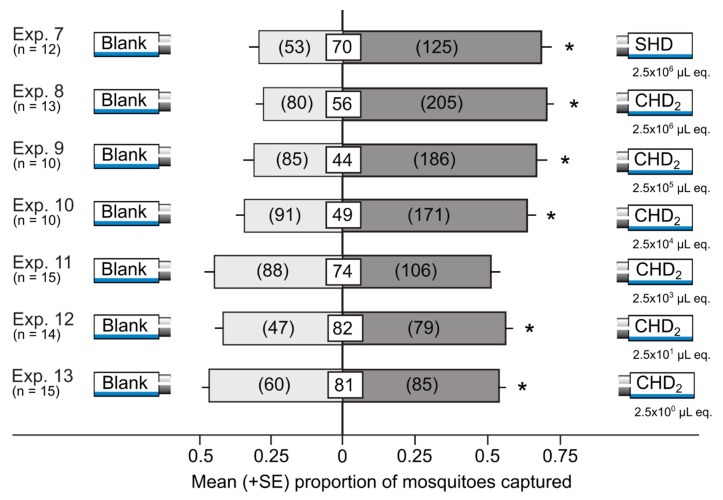
Mean proportion (+SE) of female yellow fever mosquitoes, *Aedes aegypti*, captured in Experiments 7–13 in paired traps that were baited with the SHD (a synthetic blend of sterile honeydew-derived odorants prepared according to literature data [32], Table 2) or the CHD_2_ (a synthetic blend of crude honeydew-derived odorants prepared according to literature data [32], Table 2) at descending doses or that were fitted with a corresponding solvent (blank) control. Numbers in parentheses represent the total number of mosquitoes selecting a test stimulus, and numbers within white squares indicate the mean percentage of mosquitoes not captured. An asterisk (*) indicates a significant preference for a test stimulus (*p* < 0.05; binary logistic regression). The dose of 2.5 × 10^1^ µL equivalents (eq.) of honeydew approximates the amount of honeydew produced by 25 pea aphids per day [49].

The SHD (a synthetic blend of sterile honeydew odorants prepared according to literature data [32]) at a dose of 2.5 × 10^6^ µL honeydew equivalents attracted significantly more mosquitoes than the corresponding solvent control stimulus (z = 5.2, *p* < 0.0001; Figure 4, Exp. 7).

The CHD_2_ (a synthetic blend of crude honeydew odorants prepared according to literature data [32]) attracted significantly more mosquitoes than the corresponding solvent control when tested at descending honeydew dose equivalents of 2.5 × 10^6^ μL (Exp. 8: z = 7.1, *p* < 0.0001), 2.5 × 10^5^ μL (Exp. 9: z = 6.0, *p* < 0.0001), 2.5 × 10^4^ μL (Exp. 10: z = 4.9, *p* < 0.0001), 2.5 × 10^1^ μL (Exp. 12: z = 2.8, *p* = 0.005), and 2.5 × 10^0^ μL (Exp. 13: z = 2.1, *p* < 0.039; Figure 4). Inconsistently, the CHD_2_ was not attractive at a dose of 2.5 × 10^3^ μL honeydew equivalents (Exp. 11: z = 1.3, *p* = 0.2).

**Figure 5:**
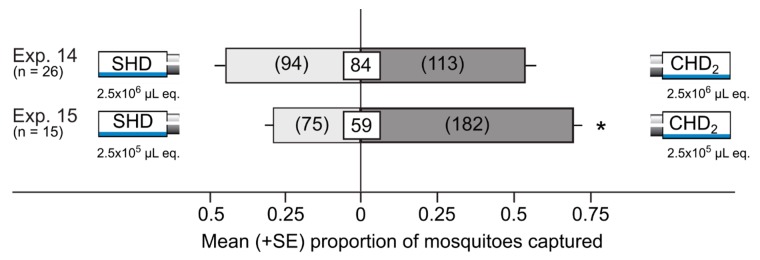
Mean proportion (+SE) of female yellow fever mosquitoes, *Aedes aegypti*, captured in Experiments 14–15 in paired traps that were baited with the SHD (a synthetic blend of sterile honeydew-derived odorants prepared according to literature data [32], Table 2) or the CHD_2_ (a synthetic blend of crude honeydew-derived odorants prepared according to literature data [32], Table 2). Numbers in parentheses represent the total number of mosquitoes selecting a test stimulus, and numbers within white squares indicate the mean percentage of mosquitoes not captured. An asterisk (*) indicates a significant preference for a test stimulus (*p* < 0.05; binary logistic regression).

When the CHD_2_ and the SHD were tested head-to-head at honeydew dose equivalents of 2.5 × 10^6^ μL (Exp. 14) and 2.5×10^5^ μL (Exp. 15), CHD_2_ at the lower dose, but not the higher dose, attracted more mosquitoes than the SHD (Exp. 14: z = 1.3, *p* = 0.2; Exp. 15: z = 6.5, *p* < 0.0001; Figure 5).

## 4. Discussion

Our data show that *Ae. aegypti* females anemotactically orient towards aphid-infested and honeydew-soiled bean plants and that synthetic blends of honeydew odorants are attractive to mosquitoes, particularly when they contain constituents of microbial origin.

Herbivory can induce the emission of plant defensive chemicals [53,54,55] that may be herbivore-specific [55] and attract natural enemies of the specific herbivore [53,54,55]. As mosquitoes were not attracted to odorants from mechanically injured plants (Figure 1, Exp. 3), or to odorants from non-feeding aphids (Figure 1, Exp. 4), it follows that mosquito females responded to either aphid-induced plant defensive chemicals that signaled aphid feeding, or to honeydew odorants. As pea aphids feeding on bean plants may not prompt the emission of plant defensive chemicals [56], it seems that the attraction of mosquitoes to plants infested with green peach aphids or pea aphids (Figure 1, Exps. 1, 2) can be attributed to odorants associated with honeydew expelled by these feeding aphids.

We present the first evidence of mosquitoes being attracted olfactorily to aphid honeydew. Our findings that honeydew from two aphid species induced the same attraction response by foraging mosquitoes suggest that honeydew odorants might be generic indicators of plant-derived sugar. The attractiveness of honeydew has previously been shown in studies with the common yellowjacket, *Vespula vulgaris* [28], the house fly, *Musca domestica* [57], and the marmalade hoverfly, *Episyrphus balteatus* [32]. Unlike hoverflies, *Ae. aegypti* females did respond to a synthetic blend of honeydew odorants lacking constituents of microbial origin (Figure 4, Exp. 1) but the dose of this synthetic blend was rather high. When we tested synthetic blends of honeydew odorants at a 10-fold lower dose, with and without the microbial odorants, mosquito females strongly preferred the more complex inclusive blend.

Some of the odorants found in natural crude honeydew may originate from the bacterium *Staphylococcus sciuri* that is known to reside in the guts of pea aphids, to metabolize honeydew, and to produce specific odorants [32]. This inference is supported by findings that the re-inoculation of sterilized honeydew with *S. sciuri* re-generated odorants typically associated with crude (non-sterile) honeydew [32]. Other odorants are likely produced by exogenous microbes that colonize and metabolize aphid honeydew over time. This would explain why freshly expelled honeydew contained only a few odorants that we could detect by GC MS analysis in our study [58]. Odorants of honeydew-dwelling microbes have been implicated in attracting the black garden ant, *Lasius niger* [59], and appear to contribute to the attraction of mosquitos to small quantities of honeydew that may otherwise not be detectable. Once mosquitoes have been attracted to and alighted on, aphid-infested plants, they can confirm the presence of honeydew via contact chemoreceptors on their tarsi [60]. Well known is that mosquitoes exploit microbe-derived odorants as resource indicators when they forage for vertebrate hosts [35,36,37,38] and select oviposition sites [39]. Here, we add to the knowledge base in that we demonstrate a role for microbe-derived odorants guiding mosquitoes to plant sugar sources.

Crude aphid honeydew seems to have common odor constituents. In crude honeydew of pea aphids feeding on fava bean plants, the same five odorants (2,3-butanedione, 3-hydroxybutanone, 3-methyl-1-butanol, 3-methylbutanoic acid, and 2-methylbutanoic acid) were found by us and a previous study [32], one odorant of which (3-methyl-1-butanol) was again just recently noted [49]. Six odorants identified here (2,3-butanedione, 3-methyl-1-butanol, 3-methylbutanoic acid, 2-methylbutanoic acid, 3-hydroxybutanone, and 2-ehtylhexanol) were also found in honeydew of black bean aphids, *A. fabae*, feeding on fava bean plants [59], and three of these odorants (2,3-butanedione, 3-methyl-1-butanol, and 3-hydroxybutanone) were noted in honeydew from vetch aphids, *Megoura viciae*, feeding on fava bean plants [27]. At least some of these odorants may originate from microbial metabolism of honeydew amino acids [49,61].

Consumption of honeydew by mosquitoes in the field [10,11] contributes to their survival [9] and is shown clearly by the presence of honeydew-specific sugars, such as melezitose or erlose, in the alimentary canal of mosquitoes [11]. However, relying solely on the presence of honeydew-specific sugars in the digestive tract of mosquitoes to gauge the extent of their honeydew consumption may lead to underestimation of this phenomenon. The constituents of honeydew change in accordance not only with the hemipteran herbivores expelling it but also the plants they feed on [62,63]. The importance of honeydew relative to floral nectar, preferential consumption of either sugar source by specific mosquito species, and the contribution of honeydew to the vectorial capacity of mosquitoes are all not yet known. Well established, however, is the view that the vectorial capacity of mosquitoes is reliant upon ready access to plant (floral) sugar [64] which is why selective removal of mosquito host plants is deemed a remedial means of shortening the longevity of mosquitoes and thus lowering their vectorial capacity [65]. This concept, however, seems to discount the effect of alternative sugar sources, such as honeydew, on mosquito longevity [9]. Like other insects [17], mosquitoes may substitute aphid honeydew for floral nectar when floral nectar is scarce or honeydew is particularly abundant [23].

## 5. Conclusions

We show that sugar-foraging females of the yellow fever mosquito are attracted to bean plants infested with green peach aphids or pea aphids. Mosquito females respond to the honeydew expelled by aphids but not to the physical presence of aphids or the mechanical damage inflicted on plants. The attractiveness of honeydew is due to its odorants. A synthetic blend of honeydew odorants tested at doses equivalent to those of honeydew-soiled plants did attract mosquitoes. At the lowest dose tested, the synthetic blend with microbial odor constituents was more attractive than the blend without these constituents. By responding to honeydew odorants, mosquitoes can locate and exploit honeydew and substitute it for floral nectar when nectar is scarce or honeydew is particularly abundant. Our study may lead to the development of a trap lure that combines mammalian-, inflorescence- and aphid-derived odorants for trapping both sugar- and blood-seeking mosquitoes.

## Figures and Tables

**Table 1:** Details of treatment and control stimuli, amount of stimuli tested, type of bioassay design, and number of replicates (N) tested with yellow fever mosquitoes in Experiments 1–15.

Exp.	Treatment ^1,2,3,4,5^	Control	Details	Design	*N*
*Attraction of Mosquitoes to Plants Aphid-Infested, Mechanically Injured, or Paired with Non-Feeding Aphids*					
1	*M. persicae*-infested *V. faba*	*V. faba*	Mean of 31 aphids per plant	Y-tubes	21
2	*A. pisum*-infested *V. faba*	*V. faba*	Mean of 103 aphids per plant	Y-tubes	22
3	*V. faba* (injured)	*V. faba*	Experimentally injured plant	Y-tubes	20
4	*V. faba* + *A. pisum*	*V. faba*	100 *A. pisum* in Petri dish	Y-tubes	22
*Attraction of Mosquitoes to Synthetic Honeydew Odorants*					
5	CHD_1_	Solvents	2.5 × 10^1^ µL honeydew equiv.	Delta traps	15
6	CHD_1_	Solvents	2.5 × 10^0^ µL honeydew equiv.	Delta traps	11
7	SHD	Solvents	2.5 × 10^6^ µL honeydew equiv.	Delta traps	12
8	CHD_2_	Solvents	2.5 × 10^6^ µL honeydew equiv.	Delta traps	13
9	CHD_2_	Solvents	2.5 × 10^5^ µL honeydew equiv.	Delta traps	10
10	CHD_2_	Solvents	2.5 × 10^4^ µL honeydew equiv.	Delta traps	10
11	CHD_2_	Solvents	2.5 × 10^3^ µL honeydew equiv.	Delta traps	15
12	CHD_2_	Solvents	2.5 × 10^1^ µL honeydew equiv.	Delta traps	14
13	CHD_2_	Solvents	2.5 × 10^0^ µL honeydew equiv.	Delta traps	15
*Attraction of mosquitoes to odorants from honeydew-dwelling microbes*					
14	CHD_2_	SHD	2.5 × 10^6^ µL honeydew equiv.	Delta traps	26
15	CHD_2_	SHD	2.5×10^5^ µL honeydew equiv.	Delta traps	15

^1^ Fava bean plants, *Vicia faba*, infested with green peach aphid, *Myzus persicae*, or pea aphid, *Acyrthosiphon pisum*; ^2^ CHD_1_: a synthetic blend of crude honeydew odorants prepared according to our own data (Figure 2 and Table 2); ^3^ SHD: a synthetic blend of sterile honeydew odorants prepared according to literature data ([32]; Table 2); ^4^ CHD_2_: a synthetic blend of crude honeydew odorants prepared according to literature data ([32]; Table 2); ^5^ Plant mechanically injured by cutting one leaf along its long axis, and then leaving the plant for 1 h prior to commencing a bioassay.

**Table 2:** Blends of synthetic honeydew odorants prepared according to compositions of crude honeydew collected in this study (CHD_1_), and in a previous study (CHD_2_) [32], and of sterilized honeydew (SHD) reported in the previous study [32].

Odorants	Purity (%)	CHD_1_ (%)	CHD_2_ (%)	SHD (%)
Propanone ^1^	99.8	-	9.25	24.62
2,3-Butanedione ^2^	86	7.70	2.31	40.54
2,3-Butanediol ^1^	98	3.49	-	-
3-Methylbutanal ^1^	97	-	14.01	-
2-Methylbutanal ^1^	>99	-	12.92	-
3-Hydroxybutanone ^1^	98	46.38	0.78	4.77
3-Methyl-3-buten-1-ol ^1^	97	-	0.89	5.64
3-Methyl-1-butanol ^3^	98.5	36.82	12.32	-
2-Methyl-2-buten-1-ol ^5^	83	-	14.41	-
3-Methyl-2-butenal ^6^	88	-	10.73	-
Butanoic acid ^1^	99	-	6.24	24.43
3-Methylbutanoic acid ^1^	99	3.07	4.56	-
2-Methylbutanoic acid ^1^	98	0.63	6.73	-
2,5-Dimethylpyrazine ^1^	99	-	0.31	-
Limonene ^1^	90	-	2.81	-
Benzeneethanol ^1^	99	-	1.73	-
2-Ethylhexanol ^1^	99	1.57	-	-
2-Phenylethyl alcohol ^4^	98	0.35	-	-

^1^ Sigma-Aldrich (St. Louis, MO, USA); ^2^ obtained by oxidation of 3-hydroxy-2-butanone; ^3^ Thermo Fisher Scientific (Waltham, MA, USA); ^4^ Fluka Chemicals Ltd. (Milwaukee, WI, USA); ^5^ synthesized by reduction of tiglic acid by lithium aluminum hydride (see Supplementary Materials; ^6^ synthesized by oxidation of 3-methyl-2-buten-1-ol by manganese dioxide (see Supplementary Materials).

## Supplementary Materials

The chemical syntheses used in this study are available online at http://www.mdpi.com/2075-4450/10/2/43/s1.
