# Supplementary material for: Attraction of Female Aedes aegypti (L.) to Aphid Honeydew
Source: Insects. 2019 Feb 1;10(2):43. doi: 10.3390/insects10020043 (PMC6409638; doi:10.3390/insects10020043)
Supplement: Supplementary file 1 [file insects-10-00043-s001.pdf]

Preparation of 3-Methyl-2-butenal:
3-methyl-2-buten-1-ol (2.82 g, 0.0327 mol) and MnO_2 (7.12 g, 0.0819 mol) were taken in petroleum ether (30 mL) and stirred at room temperature for 7 h, filtered, and the filtrate concentrated to give crude 3-Methyl-2-butenal, which was distilled off at 132°-133°C. Yield: 95% (2.612 g).

Preparation of (E)-2-Methyl-2-buten-1-ol:
Lithium aluminum hydride (3.08 g, 0.0812 mol) was added in four equal portions to ether (30 mL) at room temperature. Tiglic acid (3.02 g, 0.0302 mol) in ether (20 mL) was added dropwise over 10 min. The reaction was brought to reflux for 5 h, then cooled to room temperature and stirred for an additional 10 h. Then the reaction was cooled to 0°C, and the excess hydride was cautiously quenched by the sequential addition of water (30 mL), 15% sodium hydroxide (30 mL) and water (30 mL). The resulting white suspension was warmed to room temperature and stirred for 1.5 h, filtered through Celite, dried over magnesium sulfate, then concentrated in vacuo in a 0°C ice bath, and provided (E)-2-methyl-2-buten-1-ol as a clear, pale yellow oil, yield 94%.
